# Abnormal glucose metabolism in virus associated sepsis

**DOI:** 10.3389/fcimb.2023.1120769

**Published:** 2023-04-12

**Authors:** Peng Zhang, Shangwen Pan, Shiying Yuan, You Shang, Huaqing Shu

**Affiliations:** Department of Critical Care Medicine, Union Hospital, Tongji Medical College, Huazhong University of Science and Technology, Wuhan, China

**Keywords:** sepsis, virus, glucose metabolism, immune cell, antiviral

## Abstract

Sepsis is identified as a potentially lethal organ impairment triggered by an inadequate host reaction to infection (Sepsis-3). Viral sepsis is a potentially deadly organ impairment state caused by the host’s inappropriate reaction to a viral infection. However, when a viral infection occurs, the metabolism of the infected cell undergoes a variety of changes that cause the host to respond to the infection. But, until now, little has been known about the challenges faced by cellular metabolic alterations that occur during viral infection and how these changes modulate infection. This study concentrates on the alterations in glucose metabolism during viral sepsis and their impact on viral infection, with a view to exploring new potential therapeutic targets for viral sepsis.

## Introduction

According to the latest international guidelines for sepsis definition (Sepsis-3.0), sepsis is identified as a potentially lethal organ impairment triggered by an inadequate host reaction to infection (Singer et al., 2016). The pathogens that cause sepsis are not only limited to bacteria and fungi; viruses are also one of the pathogens that cannot be ignored. Due to the commonality of sepsis, viral sepsis follows the definition of sepsis (Lin et al., 2018). However, compared with the organ dysfunction induced by bacterial sepsis, viral sepsis has its own uniqueness, that is, the organ dysfunction caused by tissue or cell damage (such as lung epithelial damage induced by influenza virus) that is believed to be directly caused by viruses. Although there is no definitive epidemiological data on viral sepsis, studies have shown that the virus is detectable in up to 70% of patients with sepsis (Ljungstrom et al., 2017). Sepsis, which is directly caused by the virus, also occurs in up to 12% of hospitalized patients (Southeast Asia Infectious Disease Clinical Research Network, 2017). Among all viral sepsis pathogens, herpes simplex viruses, human enteroviruses, influenza viruses, dengue viruses, and others are common pathogens (Lin et al., 2018). Currently, people susceptible to viral sepsis include young children, pregnant women, the elderly, and immunosuppressed persons. Due to the uncertainties of viral sepsis, this paper reviews the epidemiology, etiology, immunopathogenesis, and potential treatment of viral sepsis.

Glucose is the main energy supply substance in animals and an important carbon source for the synthesis of macromolecular substances such as nucleic acids and lipids. Viral infection affects the absorption, transport, and metabolism of glucose in cells, providing energy and raw materials for viral gene synthesis, virion assembly, and release. We concentrated on how viral infection affects glucose metabolism in cells to better understand the etiology of viral sepsis and provide a reliable theoretical basis for future clinical treatment.

## Immune responses in viral sepsis

A viral infection immediately activates the innate immune system. Innate immune cells recognize viral infections through a variety of pattern recognition receptors (PRRs) located inside and outside the cell. These PRRs include toll-like receptors (TLRs), retinoic acid-induced gene I-like receptors (RLRs), nucleotide oligomerization domain-like receptors (NLRs), and cytoplasmic DNA sensors (Thompson et al., 2011). When confronted with pathogens, PRRs could identify pathogen-associated molecular patterns (PAMPs) and damage-associated molecular patterns (DAMPs), ultimately activating immune responses. These PAMPs include viral nucleic acids (vDNA and vRNA) and viral proteins, while DAMPs are produced by tissue damage and cell death caused by viral infection (Takeuchi and Akira, 2010; Kurt-Jones et al., 2017). In addition, activation of PRRs triggers virus-associated adaptive immunity (e.g., cytotoxic T lymphocytes, antibodies), which can eliminate cells infected with viruses to control viral infection (Iwasaki and Medzhitov, 2004). Failure to clear DAMPs released from damaged tissues and cells can lead to a more aggressive immune response and ultimately viral sepsis (Wiersinga et al., 2014).

It is commonly observed that the consequence of viral infection is to cause changes in the infected cells metabolism, along with mobilization of the body’s multi-cell collaboration to perform the antiviral function. In innate immune cells, activated PRRs and cytokine receptors can influence cell metabolism through multiple signaling pathways downstream, which are crucial in determining cell function and fate (O'Neill and Pearce, 2016). For instance, activation of TLR in conventional dendritic cells (cDCs) triggers a rapid elevation in glycolysis, which affects cytokine release and ultimately antiviral immunity (Fekete et al., 2018). Activation of TLR in plasmacytoid dendritic cells (pDC), which are the main generators of type 1 IFN, stimulates the production of IFN *via* an auto-secreted, type 1 IFN receptor-dependent pathway by inducing cellular metabolic recombination distinguished by oxidation of fatty acids and oxidative phosphorylation, thus providing antiviral activity for pDC (Wu et al., 2016). In addition, macrophages reduce the expression of 7-dehydrogen-cholesterol reductase (DHCR7) after viral infection. Deficiency of DHCR7 specifically promotes IRF3 phosphorylation and enhances type I interferon secretion (IFN-I) in macrophages, thereby regulating antiviral immunity (Xiao et al., 2020).

The immune system contains a diverse population of immune cells that are constantly in a state of dynamic proliferation and immune surveillance of the surrounding environment, and more importantly, they sustain their ability to mount a fast reaction to infections, trauma, and other triggers (Pearce and Pearce, 2013; Loftus and Finlay, 2016). These cellular functions are critical to energy requirements, and glucose, as the primary substrate for energy metabolism, provides energy for cells to carry out a variety of functions *via* two distinct mechanisms—glycolysis and the tricarboxylic acid (TCA) cycle. Glycolysis could result in the conversion of glucose to pyruvate in the cytoplasm. The TCA cycle produces nicotinamide adenine dinucleotide (NADH) and flavin adenine dinucleotide (FADH2). They undergo oxidative phosphorylation (OXPHOS) while transferring electrons to the respiratory chain. After pyruvate is converted to acetyl-CoA, glycolysis and the TCA cycle enter the common pathway of glucose metabolism (Biswas and Mantovani, 2012; Minhas et al., 2021). However, in the process of sepsis, mitochondrial damage and dysfunction are very common, which are also considered to be one of the main causes of cellular metabolic disorders. However, the shift in glucose metabolism from TCA to glycolysis increases the formation of lactate (Singer, 2014; Wasyluk and Zwolak, 2021). Furthermore, the majority of viruses can trigger the Warburg effect, which not only generates abundant energy and substrate for their replication but also increases lactic acid formation, making virus immune escape possible (Sanchez and Lagunoff, 2015; Zhou L. et al., 2021). Furthermore, the authors further explore the specific mechanism, that is, the virus achieves host-evasion of the immune defense response by enhancing hexokinase (HK) and lactate accumulation to suppress interferon release triggered by retinoic acid-inducing gene I (RIG-I) (Zhou L. et al., 2021).

The common mechanism of cell adaptation to various viral infections is the stimulation of glycolysis. During infection, viral proteins can increase the rate of cell glycolysis, which is essential for viral replication and immunity (Goyal and Rajala, 2023). Ultimately, the proliferation of viruses greatly contributes to the development of sepsis. At present, the changes in cellular metabolism that occur throughout viral infection and how they affect infection prognosis need to be further explored. In the next parts, we will go over the changes in glucose metabolism during different virus infections, which is aimed at helping us better understand the possible pathological process of viral sepsis.

## Hepatitis B virus

Hepatitis B virus (HBV) is a relaxed circular partial double-stranded DNA virus, which encodes different viral proteins such as DNA polymerase, surface antigen (HBsAg), core antigen (HBcAg), and X protein (HBx) (Zhou L. et al., 2021). This is mainly regulated by two enhancers (EnhI and EnhII) and four promoters (core, X, pre-S1, and pre-S2/S) in the genes (Shaul et al., 1985; Yee, 1989; Pang et al., 1990; Wang et al., 1990). HBV infection has a major influence on the immune cells’ glucose metabolism. The glucose metabolism disorder will have an important effect on the outcome of HBV. CD8+ T cells are essential IFN γ-mediated non-cytopathic mechanisms to clear HBV. However, in chronic HBV infection, CD8+ T cells are very few and appear to be exhausted, resulting in an obstruction of viral control (Jiang et al., 2022). Exhausted CD8+ T cells achieve metabolic recombination for glycolysis by increasing GLUT1 expression, thereby rapidly providing energy and metabolites to support proliferation and effector T-cell metabolic changes to meet antiviral requirements, although glycolysis is less energy efficient than the TCA cycle (Schurich et al., 2016), and hence interference with glycolysis may lead to T lymphocyte dysfunction (Zhou X. et al., 2021). In addition, HBV could promote HK activity and lactate dehydrogenase A (LDHA)-related lactate production, which interferes with mitochondrial antiviral signaling (MAVS). As a result of that, there is suppression of the interaction between RIG-I and MAVS, aggregation of MAVS, and induction of IFN production. These results indicate that lactate derived from HK2 and glycolysis might play an essential role in the immune escape of HBV and that recombination of glucose metabolism regulates innate immunity in HBV infection (Zhou L. et al., 2021). Although HBV infection is not uncommon, especially in China, and its effect on the glucose metabolism of infected cells is significant, sepsis caused by HBV has been rarely reported.

## Influenza virus

Influenza virus infection can have significant effects on a variety of metabolisms, including glucose metabolism, ultimately leading to the ongoing fermentation of the infection. Shortly after influenza virus infection occurs, the infected cells’ glucose uptake rate continues to increase, followed by enhanced glycolysis, leading to increased glucose consumption (Ritter et al., 2010; Petiot et al., 2011). Another glucose-consuming mechanism, the pentose phosphate pathway (PPP), is augmented by influenza infection to generate additional nucleotides, particularly ATP (Smallwood et al., 2017). These studies found a significant elevation in ATP and glucose consumption inside cells after influenza infection, as well as the reliance of the influenza virus on the glycolysis pathway for generation of energy, suggesting that the activation of the proton pump, which links glucose metabolism and ATPase, has important effects on viral replication and spread. Because inhibition of glycolysis significantly suppresses viral replication, adding ATP could re-stimulate the viral infection (Kohio and Adamson, 2013). Moreover, the increased PPP contributes to viral replication (Keshavarz et al., 2020). Besides, infection with the influenza virus could decrease glucose-6-phosphate dehydrogenase (G6PD) expression and activity, which exacerbates oxidative stress and replication (De Angelis et al., 2021). Furthermore, G6PD downregulation was associated with a reduction in nuclear factor erythroid 2-related factor 2 (NRF2), which modulates the antioxidant response gene network during influenza virus infection (De Angelis et al., 2021). It reveals a new approach deployed by the influenza virus to trigger oxidative stress and replication. These findings demonstrate that modification of metabolic mechanisms can reveal novel therapeutic strategies to combat influenza virus infection (Ohno et al., 2020).

Elevation of glucose uptake and glycolysis might be linked to an imbalance of the PI3K/AKT/mTOR signal pathway, which at last influences c-Myc expression in the infected cells (Smallwood et al., 2017). A range of influenza virus proteins can stimulate the mechanistic targets of rapamycin complex 1 (mTORC1) and mTORC2, which promote c-Myc expression (Wall et al., 2008; Masui et al., 2013). Myc promotes glycolysis by overexpressing of the glucose transporter GLUT1, glycolytic genes, and lactate dehydrogenase (LDH), which converts pyruvate to lactate (Gordan et al., 2007). MTORC1 is also involved in the upregulation of hypoxia-inducible factor-1α (HIF-1α), a factor that stimulates a number of gene expressions, including many glycolytic enzymes, glucose transporters, and LDH (Duvel et al., 2010; Semenza, 2010). AKT can increase glycolysis by suppressing FoxOs (Bloedjes et al., 2022). AKT can stimulate GLUT1 expression and membrane localization, as well as phosphofructokinase action (Barthel et al., 1999). Furthermore, mTORC1 enhances the expression of G6PD, a rate-limiting enzyme in the oxidative part of PPP (Duvel et al., 2010).

When comparing glycolysis and PPP, a small amount of glucose is metabolized by the hexosamine biosynthesis pathway, producing uridine diphosphate N-acetylglucosamine (UDP-GlcNAc) (Hardiville and Hart, 2014). O-linked β-N-acetylglucosamine (O-GlcNAc) transferase (OGT) facilitates the transfer of UDP-GlcNAc to target protein serine (S) or threonine (T) residues, a process known as protein O-GlcNAcylation (Yang and Qian, 2017). Influenza A virus (IAV) stimulated OGT to be attached to interferon regulatory factor-5 (IRF5), resulting in O-GlcNAcylation of IRF5, which is required for IRF5 ubiquitination related to K63 and additional downstream inflammatory cytokine secretion. It also revealed a molecular mechanism through which IRF5 activity during IAV infection is modulated by HBP-mediated O-GlcNAcylation, highlighting the impact of glucose metabolism in IAV-induced cytokine storms (Wang et al., 2020). Influenza virus infection causes respiratory diseases and remains a major health concern. It can increase viral replication by enhancing glucose uptake and glycolysis, as mentioned above, and blocking the pathways involved in glucose metabolism may benefit the disease caused by influenza infections.

## Dengue virus

The dengue virus (DENV) is a plus-stranded RNA virus from the Flaviviridae family. It predominantly causes dengue fever, dengue hemorrhagic fever, and dengue shock syndrome and is spread by the *Aedes aegypti* and *Aedes albopictus* vectors. Patients and recessively infected individuals are the main sources of infection. Studies have demonstrated that abnormal glucose metabolism, particularly the glycolysis pathway, is essential for enhancing viral replication during DENV infection (Al-Alimi et al., 2014; Fontaine et al., 2015; Fernandes-Siqueira et al., 2018). First, mosquitoes (*Ae. aegypti* UGAL/Rockefeller strain) were orally infected with DENV (serotype 2, 16681 strain) through infectious blood feeding; a significant increase in DENV genome levels in mosquitoes consuming an infectious blood meal supplemented with glucose could be found, revealing that high glucose can promote viral replication in a way that depends on the AKT and TOR signaling pathways (Weng et al., 2021). Then, DENV2 infection regulates glucose metabolism at the post-transcriptional level in an independent lactate pathway. This is because in DENV2-infected cells, glucose uptake and levels of glucose transporter 1 (GLUT1) expression and Hexokinase 2 (HK2), the glycolytic rate-limiting enzyme, are elevated. However, ATP levels were reduced with no influence on HK2, expression of phosphofructokinase mRNA, or lactic acid production. In addition, autophagy has been shown to likely be involved. These studies may provide future guidance for the treatment of DENV by targeting metabolic regulation (Lee et al., 2020). DENV infection can disrupt the ability of immune cells, including mast cells, dendritic cells, monocytes, macrophages, and T and B cells, to defend against infections, thereby exacerbating viral replication and transmission (Khanam et al., 2022). Monocytes deficient in G6PD (glucose-6-phosphate dehydrogenase) exhibited a significant elevation in the rate of DENV2 infection. Both nitric oxide (NO) and superoxide anion (O_2_^−^) levels were significantly decreased, and overall oxidative stress was significantly higher. As a key enzyme in the pentose phosphate pathway, G6PD has a major impact on glucose metabolism. DENV can also activate glycolysis, and inhibition of glycolysis can significantly block the production of DENV-infected cells. However, at present, effective vaccines or specific antiviral therapies against DENV are still in the clinical and experimental stage, and future exploration based on glucose metabolism in immune cells is expected to provide opportunities for dengue infection control (Al-Alimi et al., 2014).

## Cytomegalovirus

Human cytomegalovirus (HCMV) is a widespread herpesvirus. HCMV infection can cause dramatic changes in glucose metabolism. Overall, HCMV infection leads to an increase in glycolysis efficiency. From the mechanism, firstly, this is due to the significant upregulation of the adipose tissue-specific glucose transporter type 4 (GLUT4) expression, which increases glucose uptake (Rodriguez-Sanchez and Munger, 2019). In addition, after HCMV infection, calcium/calmodulin-dependent kinase (CaMKK) is triggered (McArdle et al., 2011; McArdle et al., 2012). As an upstream calc-calmodulin cascade kinase, it leads to AMPK kinase activation and subsequent glycolysis upregulation (Woods et al., 2005). Activated AMPK in turn stimulates glycolysis by GLUT4 (Yu et al., 2011). Infection is amplified through the interaction of the appeal mechanism (CaMKK is activated after HCMV infection; it leads to AMPK kinase activation and activated AMPK in turn stimulates glycolysis by GLUT4). Based on appeals, it was recently found that overexpression of downstream GLUT4 did not directly restore viral replication when upstream CaMKK was inhibited. However, when AMPK is inhibited, HCMV replication is partially saved, emphasizing the GLUT4 transporter’s role in AMPK-mediated HCMV infection. In summary, glycolytic activation is a major part of HCMV infection, and further exploration of how HCMV reprograms cell metabolism is beneficial to reveal potential viral treatment options (Dunn et al., 2020).

Because of its capability to escape immune surveillance at several levels, HCMV can survive in the host. IFN-inducible protein 16 is a major player in this process (IFI16) (Gariano et al., 2012). However, IFI16 downregulates GLUT4 transcriptional activation by interaction with carbohydrate response element binding protein (ChREBP), reduces the transcription of HCMV-induced lipogenesis enzymes, which in turn reduces uptake and consumption of glucose, decreases lipid synthesis, and, ultimately, prevents the formation of new viral particles. It provides new prospective approaches to the research and development of antiviral drugs (Griffante et al., 2022).

Immune cells, as the main performers of antiviral cells, also have significant changes in glucose metabolism in the viral microenvironment. Cytomegalovirus infection changes the glucose metabolism of NK cells, thus altering their antiviral activity and leading to prolonged viral infection in the body (Mah et al., 2017). However, there are few studies on cytomegalovirus glycometabolism in other immune cells at present. Future studies may focus on the changes and regulation of glycometabolism to provide possible therapeutic drugs for viral immunity.

## SARS-CoV-2

COVID-19 is a highly contagious viral disease that has attracted people’s attention because of its serious harm. It is currently known to be caused by acute respiratory syndrome coronavirus 2 (SARS-CoV-2). Altered glucose metabolism is also a common phenomenon during COVID-19. This is because new hyperglycemia does not appear to be a rare phenomenon in people with COVID-19 who have never had diabetes. This impact appears to be mediated by the abnormal secretome, which is still mutated even after disease recovery. IL-6 plays an important role in the proinflammatory environment triggered by a cytokine storm. It induces insulin resistance and beta-cell dysfunction, leading to clinically evident hyperglycemia that is detectable even after the acute phase of COVID-19 (Montefusco et al., 2021). Fasting glucose level elevations were identified as a risk factor after an acute COVID-19 infection (de Arriba et al., 2022); also, admission hyperglycemia and deteriorating blood glucose were strongly related to severe COVID-19 risk factors, and deteriorating blood glucose may be more probable to occur in COVID-19 (Xiao et al., 2022). SARS-CoV-2 can invade and replicate in human islet culture, and infection causes morphological, transcriptional, and functional alterations, such as decreased insulin-secretory granules in β cells and impairment of glucose-stimulated insulin secretion. It is recognized that SARS-CoV-2 infection targets the human pancreas and proposed that β-cell infection could play a part in the metabolic abnormalities found in COVID-19 patients (Muller et al., 2021).

Targeting the metabolism of infected host cells as a technique to stop viral development and spread in infected hosts is supported by recent clinical studies with 2-DG in SARS-CoV-2-infected individuals (Pajak et al., 2022). Because glycolysis and glutaminolysis are required for virus replication, metabolic disruption of these processes can prevent SARS-CoV-2 replication and can also be an effective antiviral approach (Krishnan et al., 2021). GP73 is a type II transmembrane Golgi protein found on the luminal side of the Golgi apparatus. In one study, GP73 release was elevated in cells infected with SARS-CoV-2 (Wan et al., 2022). GP73 can enhance fasting glycemia by stimulating hepatic gluconeogenesis *via* the cAMP/PKA pathway. According to these results, GP73 is a glucose-stimulating hormone that may cause systemic glucose metabolism abnormalities induced by SARS-CoV-2 and neutralize plasma GP73 may be an effective means of treating SARS-CoV-2 (Wan et al., 2022).

During viral infection, mononuclear phagocytes such as monocytes and macrophages play a crucial role in the innate immune system, releasing pro-inflammatory cytokines (Nikitina et al., 2018; Vangeti et al., 2018; Pence, 2020). Monocytes treated with recombinant spike protein subunit 1 from SARS-CoV-2 encounter a dose-dependent elevation in glycolysis, which is inhibited by a hypoxia-inducible factor-1a (HIF-1a) inhibitor and facilitates the generation of pro-inflammatory cytokines. IL-6 protein expression was elevated in SARS-CoV-2 infected monocytes but was suppressed by metformin pretreatment. Metformin reduced the secretion of cytokines and markedly inhibited glycolysis and cellular respiration, indicating that it could be a possible therapeutic protocol for COVID-19 hyperinflammation (Cory et al., 2021). In CoV-2-infected monocytes, 2-DG treatment provided full prevention of viral replication and CoV-2-enhanced elevations in angiotensin-converting enzyme 2 (ACE2) and IL-1β expression. HIF-1a is a strong inducer of glycolysis, and IL-1β and reactive oxygen species (ROS) are strong inducers of HIF-1a. HIF-1a inhibition blocks the expression of ACE2, IL-1β, TNF-a, IL-6, and IFNα, β, and λ, which have been implicated in the COVID-19 cytokine storm. MitoQ or NAC shows effectiveness in inhibiting viral replication as well as CoV-2-induced HIF-1a stabilization and ACE2 and the expression of IL-1β in CoV-2-infected monocytes. The study provided mechanistic proof that the mitochondrial ROS/HIF-1a/glycolysis axis could be a target for treatment of COVID-19 disease (Codo et al., 2020). As an emerging crisis, SARS-CoV-2 has become a major global public health threat in recent years. Although there is no specific drug at present, the prognosis of patients can be greatly improved by the management of blood glucose and the regulation of glucose metabolism during COVID-19 infection.

## Drugs

Currently, there are limited effective drugs for viral infections in sepsis. Drugs that affect glucose metabolism to treat viral sepsis are still in preclinical research. Although metformin was widely used to control blood glucose in the early days, current studies suggest that metformin may play a role in infectious diseases, which may extend its therapeutic role in sepsis viral infection (Malik et al., 2018). Because metformin inhibits glycerol-3-phosphate dehydrogenase, increasing the cytoplasmic redox state and ultimately inhibiting hepatic gluconeogenesis, it inhibits a variety of viruses, including hepatitis B (LaMoia and Shulman, 2021). As mentioned above, metformin can also play a role in inhibiting SARS-CoV-2 by regulating the proinflammatory immunometabolism response of monocytes (Codo et al., 2020). 2-deoxyglucose (2-DG) and its novel analogs can exert antiviral effects by regulating the glycolytic pathway of infected cells (Codo et al., 2020). However, inhibition of glycolysis by 2-DG can lead to systemic metabolism shifting to lipid catabolism, which may increase the severity of ARDS. This brings uncertainty to clinical use (Nolan et al., 2021). It has been confirmed that abnormal blood glucose levels, especially hyperglycemia, promote viral replication (Shen et al., 2023), and further studies are warranted to better understand the complex mechanisms underlying these efficacy and effects. Ongoing studies on the regulation of glucose metabolism and reorganization may provide possible molecular targets for the treatment of viral sepsis.

## Conclusions

**Figure 1:**
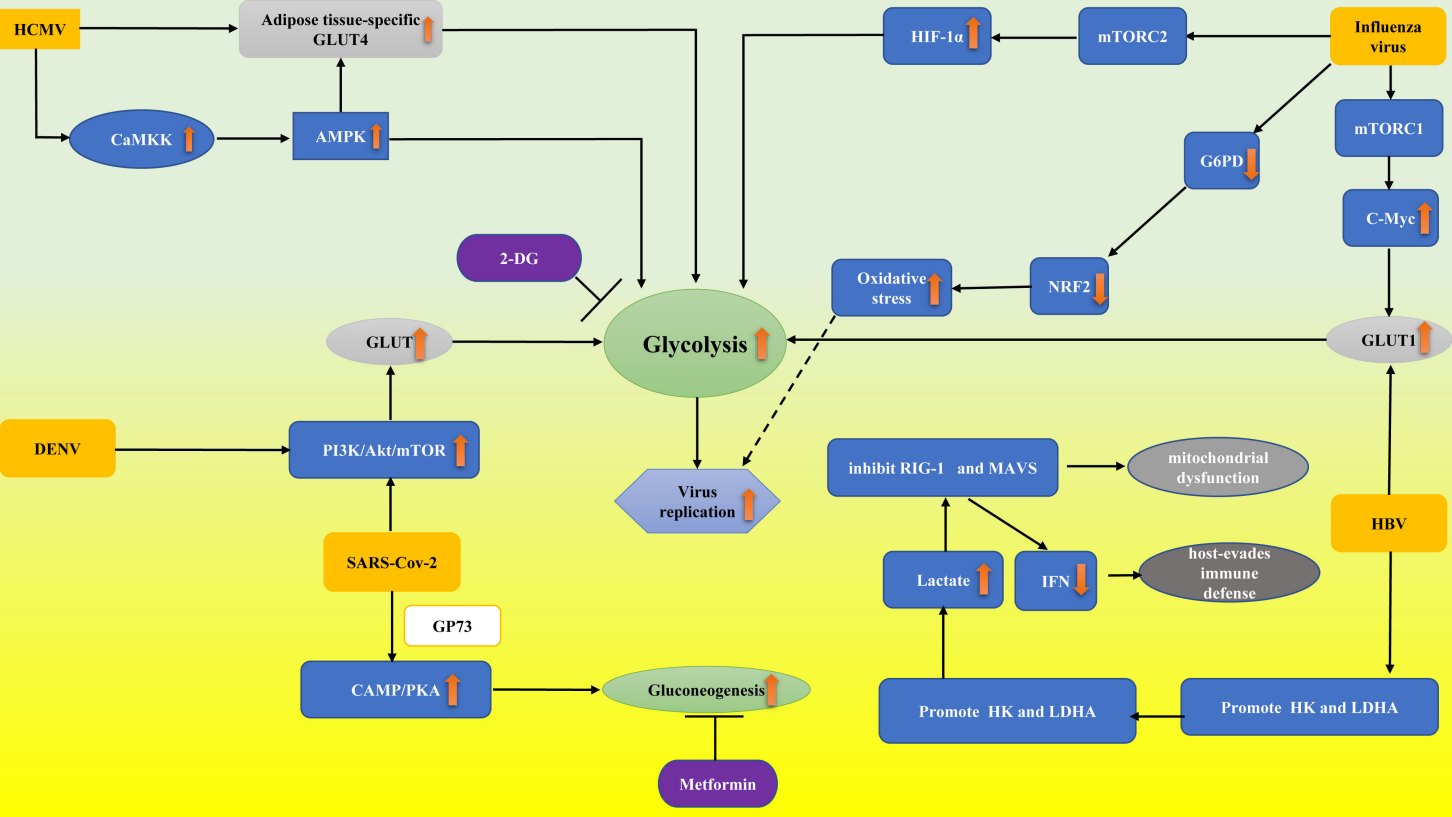
Abnormal glucose metabolism in virus-associated sepsis. Influenza virus decreased the expression and activity of G6PD and thus downregulated the expression of NRF2, which regulates the gene network of the antioxidant response. Finally, the oxidative stress and virus replication were aggravated. Influenza virus proteins promote glycolysis by stimulating the mTORC1 and mTORC2 pathways by upregulating GLUT1 and HIF-1α expression, respectively. DENV activated the PI3K/Akt/mTOR pathway to upregulate GLUT expression, thus enhancing glycolysis. HCMV resulted in AMPK activation through CaMKK. AMPK activation resulted in glycolytic activation to provide energy and facilitate virus replication. HCMV infection can upregulate GLUT4, which increases glucose uptake. Activated AMPK in turn stimulates glycolysis by GLUT4. HBV achieves glycolytic metabolic recombination by increasing GLUT1 expression. HBV regulates HK activity, and LDHA stimulates lactic acid production, thereby inhibiting RIG-I interaction with MAVS and leading to immune escape by regulating IFN production. SARS-CoV-2 activated the PI3K/Akt/mTOR pathway and led to the release of GP73, which stimulated liver gluconogenesis to enhance fasting glucose through cAMP/PKA. 2-DG and Metformin inhibit viral replication through the mechanisms of suppressing glycolysis and gluconeogenesis, respectively. PI3K/Akt/mTOR pathway, phosphatidylinositide 3-kinases/protein kinase B/mammalian target of rapamycin; cAMP/PKA, cyclic adenosine monophosphate/protein kinase A; CaMKK, calcium/calmodulin dependent kinase; NRF2, nuclear factor erythroid 2-related factor 2; G6PD, glucose-6-phosphate dehydrogenase; 2-DG, 2-deoxy-D-glucose; MAVS, mitochondrial antiviral signaling; HK, hexokinase; RIG-I, retinoic acid-inducing gene I; HIF-1α, hypoxia-inducible factor-1α; mTORC1, rapamycin complex 1; GP73, a type II transmembrane Golgi protein; GLUT4, adipose tissue-specific glucose transporter type 4. The upward orange arrow means upregulation.The downward orange arrow means downregulation.

At present, viral sepsis is underdiagnosed and heterogeneous for various reasons, which brings great difficulties for clinical diagnosis and treatment. According to the previous review, viral infection can directly or indirectly interfere with the glucose metabolism of infected cells in a variety of ways. Although many research results have not been confirmed in immune cells, as direct participants in antiviral therapy, glucose metabolism may be significantly reprogrammed in the viral microenvironment, which provides interference pathways for the regulation of antiviral immunity. In this study, we outlined the involvement of glucose metabolism in several viral infections (**Figure 1**) (such as lactate produced by glycolysis playing a major role in HBV immune escape; HBP-mediated O-GlcNAcylation controls IRF5 function during IAV infection, emphasizing the glucose metabolism role in IAV-induced cytokine storm; targeting the mitochondrial ROS/HIF-1a/glycolysis axis can prevent “cytokine storm” in COVID-19 disease). Viruses, especially SARS-CoV-2, pose great challenges to us. To fully understand the changes in immune cells glucose metabolism from the viral perspective and to explore the signaling pathways and intermediates involved, one will not only participate in a better understanding of pathophysiological mechanisms but will also possess potential for the creation and application of novel vaccines or therapies in the future.

## Author contributions

PZ and SP made equal contribution to this work and wrote the manuscript. HS and YS revised the manuscript. All authors contributed to the article and approved the submitted version.
